# The Book World of Medicine and Science

**Published:** 1904-10-22

**Authors:** 


### The Book World of Medicine and Science.

Municipal Shortcomings: The Example of Liverpool. A Series of Articles contributed to the *Liverpool Journal of Commerce* by T. Myddleton Shallcross. (London: Elliot Stock, 62 Paternoster Row, E.C. Price 1s. net.)

Mr. Shallcross's title is less wide than his subject, for he deals not merely with municipal shortcomings, but with faults of conduct, great and small, for which it is rather futile to blame the Municipality of Liverpool or of any other town. He condemns, for example, the wearing by ladies of long dresses, which, when not carefully held up, sweep up the foul dirt of the streets. That infection may be thus conveyed is undeniable, but he would be a brave Lord Mayor who would try to revive the sumptuary laws of the Middle Ages, even in the cause of sanitation. In short, Mr. Shallcross in many of his articles confounds the individual with the community, and demands that the latter shall exercise an altogether impossible control over the former. But, beyond this criticism, we find in Mr. Shallcross's brochure much to praise, and his comments on the doings and misdoings of the Liverpool Municipality would apply with equal force to many other towns. Liverpool is inclined to do in the interests of public health great and expensive things, while ignoring those smaller and cheaper improvements which are equally important. He gives us examples: "On the one hand, costly schemes for creating and laying out open spaces; on the other hand, existing open spaces permitted to be built upon. On the one hand, elaborate administrative machinery for the disposal of refuse; on the other hand, pollution of streets and atmosphere in the process of its removal. On the one hand, costly hospitals and healing staff for those who have become ill; on the other hand, neglect to enforce simple precautions to maintain the health of those who are well, and to avoid accidents happening to them." The obvious explanation of this is that even the best-intentioned City fathers, if they lack scientific knowledge, are not alert to the possibilities of disease and danger, and require actual proof of them before they will undertake remedial measures. Indeed, it is doubtful if the ideal Corporation, which Mr. Shallcross would like, has the remotest chance of being elected in even the most progressive city. But when Mr. Shallcross takes his municipality to task for not trying to prevent admitted dangers, he certainly has right on his side. The risk to life and health from eating unsound and adulterated food is well known, yet how little energy seems to be employed in putting into force the existing Adulteration Act! Thus the Medical Officer of Health for Liverpool reports that nearly 120 tons of meat and 54 tons of fish had been condemned as unfit for human food during the year 1902; but for this none of the offenders was sent to prison, and only nine were fined, the amount of the fines and costs being £81 10s., something less than £10 a head. If is doubtful if such treatment will act as a deterrent. In fact, while it is highly dangerous to poison anyone by means of drugs, it does not seem to be a serious crime in the eyes of the law to poison by means of unsound food. The chances are that the murderer will escape detection altogether and make a handsome profit, and at the worst he will get off with a £10 fine. As a matter of fact, there is often a good deal of sympathy felt for those who are caught, on the ground that they did not know that their wares were unfit for human food, but it is almost certain that even in these cases severer penalties would have a marked educational influence. Even more trivial is the punishment meted out to those who are responsible for overcrowded and insanitary workrooms. In the year 1902 the number of workshops found to be incorrect or defective was 3,458, and the amount received in fines from the owners of these amounted to what Mr. Shallcross calls "the truly magnificent sum of £2 9s. 6d!" When we consider the effect on the health of the community which these insanitary workrooms must cause, we must agree that the Liverpool sweaters are a favoured race. With regard to housing improvement, Mr. Shallcross's summary of the work of the Liverpool Corporation holds good of many other towns also. He says that "It seems to be to pay owners of property which is situate within insanitary demolition areas for the right of the Corporation to remove such property, and then for the Corporation to replace it at the expense of the ratepayers by even smaller-roomed dwellings; substituting in fact blocks of vertical overcrowding for horizontal congestion." We hope that in this he does an injustice to the Corporation, but in view of the fact that the percentage of single-roomed tenements is equal to 6·1 per cent. of the whole, it must be admitted that the housing question in Liverpool seems to be as yet a long way from solution. It is only just to say that Mr. Shallcross does not content himself with finding fault with the existing state of things and with those whom he regards as responsible for them. He advocates the formation of a Citizens' Union, which he hopes might do something "towards arousing an apathetic public and slowly moving authorities to an appreciation of existing facts, of the necessity of remedial measures to combat known evils, and of the possibility of successfully solving even difficult problems." There is no doubt that such an association would strengthen the hands both of officials and of the municipality, who always find it difficult to act much in advance of public opinion. To all who are interested in the health of our towns this little book will be helpful and suggestive.

Indian Public Health. A Journal for the Discussion of Public Health Questions. Edited by Dr. A. G. Newell, D.P.H. (Calcutta: Issued monthly. Annual subscription Rs. 12.)

In his preface to the initial number of this journal the editor calls attention to the fact that millions of people in India die every year simply through the neglect of the very rudiments of hygiene. What little sanitary virtue exists is concentrated in the religious rites of the various sects, and hardly finds its way into the everyday life of the general population, simply because its rationale is not understood. What the nation of India requires therefore is education in sanitary matters, and Dr. Newell is hopeful that the periodical which he is nurturing may go some little way to filling this public want, and we heartily commend the spirit of his enterprise, and wish it all success. The most noteworthy articles in the number before us are those on Road Sanitation, Insanitary Houses and Areas and their Relation to Plague, the Prevention of Malaria, and Medical Administration in Egypt.

